# Impact of SARS-CoV-2 Pandemic on the Diagnosis of Cervical Cancer and Precursor Lesions—A Single-Center Retrospective Study

**DOI:** 10.3390/medicina60060909

**Published:** 2024-05-30

**Authors:** Lavinia Balan, Cristina Secosan, Virgiliu-Bogdan Sorop, Marilena Pirtea, Anca Maria Cimpean, Daniela Chiriac, Catalin Balan, Ema Borsi, Ariana Iorga, Laurentiu Pirtea

**Affiliations:** 1 Department of Obstetrics and Gynecology, Victor Babes University of Medicine and Pharmacy, 300041 Timisoara, Romania; lavinia.balan@umft.ro (L.B.); bogdansorop@yahoo.com (V.-B.S.); marilena.pirtea@umft.ro (M.P.); danielachiriac89@yahoo.com (D.C.); pirtea.laurentiu@umft.ro (L.P.); 2 Department of Microscopic Morphology/Histology, Victor Babes University of Medicine and Pharmacy, 300041 Timisoara, Romania; acimpeanu@umft.ro; 3 Department of Cell and Molecular Biology, Victor Babes University of Medicine and Pharmacy, 300041 Timisoara, Romania; bcatalin43@yahoo.com; 4 Department of Internal Medicine, Discipline of Hematology, Victor Babes University of Medicine and Pharmacy, 300041 Timisoara, Romania; ema.borsi@umft.ro; 5 Clinical Hospital of Infectious Diseases and Pulmonology “Dr. Victor Babes”, 300310 Timisoara, Romania; ariana.iorga.umftvb@gmail.com

**Keywords:** COVID-19, SARS-CoV-2, cancer diagnosis, conization, cervical cancer, colposcopy, dysplasia, biopsy

## Abstract

*Background and Objectives*: Our aim was to perform a retrospective analysis of the volume of cervical screening tests, the number of patients treated with an excision method, and the incidence of invasive and non-invasive cervical during a pandemic and pre-pandemic period of 24 months. *Materials and Methods*: The study compared 404 patients who underwent cervical cone biopsy for cervical cancer. The study examined patients’ specimens based on histopathological characteristics and categorized cervical lesions based on pap smear. *Results*: There was a statistically significant age difference between the two study periods. The mean difference was 32 years before the pandemic and 35 years during the pandemic (*p*-value > 0.05). The biggest patient loss ratio identified by age group was in the 50–59-year group, with a 14.53% loss in the pre-pandemic period and a 9.1% loss in the pandemic period. In the pandemic period, patients from rural areas presented in the clinical trial with a lower rate of 39.52% (83 patients) vs. 60.47% (127 patients) in urban areas. A higher percentage of patients experiencing cervicorrhagia as a clinical manifestation in the pandemic period vs. the pre-pandemic period, with an increase in more severe lesions in the pandemic period, had a statistical significance of 8% more newly diagnosed compared to the pre-pandemic period. *Conclusions*: The addressability of the patients during the COVID period was not affected in a drastic way in our study. We encountered a decrease in appointments in the age group of 50–59 years and a decrease in patients with rural residence. In our study, we found an increase in cervical bleeding as a reason for consultation in the pandemic period with a higher lesion degree, both on a pap smear and on a cervical biopsy.

## 1. Introduction

Cervical cancer is the fourth most common type of cancer in the world after breast, colorectal, and lung cancer. According to the GLOBOCAN (Global Cancer Statistics) report for 2020, there were an estimated 604,127 new cases and 341,831 deaths worldwide. In Europe, 58,169 new cases and 25,989 deaths were estimated in 2020 [1]. All countries are affected, but incidence and mortality are higher in low- and middle-income countries; many of these countries do not have effective population-based screening programs [1,2,3]. Worldwide, cervical cancer remains the second most prevalent cause of mortality due to cancer among women aged from 20 to 39 [4]. In 2018, it was estimated that there were approximately 569,847 new cases of cervical cancer [5]. Studies have shown that cervical cancer can be a largely preventable disease [6]. By screening (particularly with HPV-based methods) and early detection by up to 80% [7], and with vaccination of the population against the most oncogenic human papilloma virus (HPV) types [8], cervical cancer can be eradicated [9].

The prevalence of cancer has shown a decline in developed nations, primarily due to lifestyle modifications such as smoking cessation, enhanced screening methods, and improved treatment options. However, low-income and developing countries have experienced a general rise in both cancer mortality and incidence rates [10].

The state of this disease in Romania is quite concerning. According to the International Agency for Research on Cancer for 2020, in Romania, cervical cancer is the third most common type of cancer in women, with a mortality rate of 11.23/100,000. Romania has the highest mortality rate for cervical cancer in the European Union (EU). The average mortality rate in the EU from this type of cancer is 3.4 per 100,000 inhabitants, while in Romania, around 11 women per 100,000 die each year from this disease [11]. Of all cervical cancer cases diagnosed in the EU, 7.5% come from Romania, a rate three times higher than the EU average. Romania occupied the second position in Europe after Montenegro [12].

Cervical cancer is a common condition, with the squamous variant slowly progressing over time in most patients. The long-time interval to advance a cervical intraepithelial neoplasia (CIN) 1 lesion to a CIN 3 lesion, the increased frequency of cases, as well as the existence of an excision-type treatment that allows healing of the initial stages, are arguments for regular cervical cancer screening [13]. The introduction of the pap smear in 1940 revolutionized the detection of morphological alterations in the cervical epithelium, resulting in a notable decline in the occurrence and fatality rates of cervical cancer in developed nations. In our time, the pap smear remains the fundamental diagnostic tool in the majority of contemporary screening programs [13]. Because cervical premalignant diagnosis and screening intensity have decreased as a result of the coronavirus disease 2019 (COVID-19) pandemic, a new population of vulnerable women has emerged [1,14,15]. Severe acute respiratory syndrome (SARS-CoV-19) infection poses a serious threat to people with underlying chronic conditions, including cancer. In the early stages of the COVID-19 pandemic, there was a sharp decrease in the detection of various types of cancer [16,17], with an increased proportion of cancer diagnoses at advanced stages [18]. This negative effect was not only due to shortcomings in health systems, a lack of preparedness, and resource availability but also to the increased number of COVID-19 cases, where the demand for SARS-CoV-2 testing is competing with the ability to deliver HPV testing [18], compounded by a shortage of staff [19].

Additionally, the pandemic has led to a widespread deferral of elective procedures, including cancer screenings and surveillance colonoscopies, which are critical for the early detection and management of oncological diseases [20]. Furthermore, there has been a noticeable disruption in the continuity of care for cancer patients, as many have experienced postponed surgeries or altered chemotherapy protocols, impacting patient outcomes [21]. The interruption of clinical trials during the pandemic has also raised concerns about the delay in the development and approval of new cancer treatments [22]. Moreover, the implementation of social distancing measures and the fear of contracting the virus in healthcare settings have caused many patients to avoid or delay seeking medical attention for cancer-related symptoms, potentially leading to more severe health conditions upon eventual presentation [23]. The psychosocial impact on cancer patients, already vulnerable due to their condition, has been profound, with increased reports of anxiety, depression, and other mental health issues as they face heightened uncertainty and isolation [24]. The long-term implications of these pandemic-related disruptions on cancer care outcomes are yet to be fully understood, but early data suggest a significant impact on survival rates and quality of life for cancer patients worldwide [25].

The aim of the current study was to analyze the prevalence of patients admitted with different types of cervical dysplasia and conization treatment during the two years before the pandemic in comparison with the period of the SARS-CoV-2 pandemic. We performed a retrospective analysis of the volume of cervical screening tests, the number of patients infected with HPV, the number of patients treated with an excision method, and the incidence of invasive and non-invasive cervical lesions.

## 2. Materials and Methods

### 2.1. Design and Ethics

The study was conducted according to the guidelines of the Declaration of Helsinki and approved by the Ethical Committee of the Municipal Emergency Clinical Hospital of Timisoara number 81/12.12.2022. This study followed a retrospective design, and all patient data have been anonymized.

We collected data and compared the number of 404 patients who underwent cervical cone biopsy in the Department of Obstetrics and Gynecology of the Timisoara Clinical Municipal Hospital for cervical cancer during the pandemic (210 patients) and the pre-pandemic periods (194 patients). The inclusion criterion consisted of patients with an indication for conization during the pandemic period. We excluded patients with previous diagnoses of cervical cancer who were hospitalized for radical surgical treatment, or those who underwent hysterectomy, for being outside of our study’s focus. Also, the study did not follow the patients included in the study. We performed a retrospective analysis of the volume of cervical screening tests, the number of patients infected with HPV, the number of patients treated with an excision method, and the incidence of invasive and non-invasive cervical lesions.

### 2.2. Analysis of Samples

Primary processing of surgical biopsies: The tissue samples were fixed in 10% buffered formalin for 24–48 h before being paraffin embedded using all of the methods necessary to generate paraffin blocks formalin-fixed paraffin-embedded (FFPE). For each example, we chose one block that we thought was relevant for our study.

The methodology of this study entailed the examination of patients’ specimens based on their histopathological characteristics. All specimens obtained from surgical procedures were processed and examined in a pathological anatomy laboratory specialized in histopathology. The process involved the application of hematoxylin and eosin stains to the tissue fragments to ascertain the degree of lesion severity.

To evaluate the outcomes of the study, a comprehensive analysis of various parameters was conducted. These parameters included the age and medical history of the patients, the childbearing potential or menopausal status, and the presence or absence of cervical bleeding, which is abnormal cervical bleeding, which was defined as cervical bleeding at the time of clinical examination and noted as having been present or absent from the patient’s clinical assessment at the time of the collection of samples for the pap smear. Additionally, the types of cervical lesions were categorized based on the findings from pap smears. We also analyzed the results of the HPV molecular diagnostic tests, which provided insights into the presence of one or multiple HPV strains, including those with high oncogenic potential.

The study further investigated the number of patients who underwent colposcopy and conization. Women had one cervical sample collected into a liquid-based cytology vial (ROCHE, Heidelberg, Germany) using either a spatula or brush. Immunocytochemistry analysis was performed using the CINtec PLUS Cytology kit (Roche MTM Laboratories, Heidelberg, Germany), according to the manufacturer’s instructions on the cervical pap smear sample. HPV genotyping and dual staining for p16/Ki-67 were performed prior to LEEP in selected cases. The dimensions of the conization specimens, which involved the removal of a cone-shaped section of abnormal tissue from the cervix, were meticulously recorded. In cases where a concomitant endocervical biopsy was performed using curettage, this information was also captured. Finally, the histopathological diagnosis established the definitive nature of the cervical lesions.

HPV genotyping was performed by the LINEAR ARRAY HPV Genotyping Test [CEIVD] (ROCHE, Heidelberg, Germany). The HPV test is based on the amplification of target DNA (HPV L1 gene) by multiplex PCR (polymerase chain reaction) and reversed hybridization of the amplified products to a linear array of 37 immobilized probes, which represent different HPV types (6, 11, 16, 18, 26, 31, 33, 35, 39, 40, 42, 45, 51, 52, 53, 54, 55, 56, 58, 59, 61, 62, 64, 66, 67, 68, 69, 70, 71, 72, 73, 81, 82, 83, 84, IS39, and CP6108). PCR was performed on a GeneAmp PCR System 9700 thermal cycler (Applied Biosystems, Waltham, MA, USA), according to the manufacturer’s instructions. Automated hybridization and detection of HPV-DNA was performed on a ProfiBlot 48 Western Blot processor (Tecan Trading AG, Zurich, Switzerland).

### 2.3. Statistical Analysis

Statistical analysis was conducted using Excel 2021 and JASP 0.18.3 software. We initially applied an F-test to assess the equality of variances between the two groups, a necessary step before utilizing T-tests for comparison of patient groups in the pre-pandemic and pandemic periods. Fisher’s exact test was used to compare proportions between study groups. Additionally, linear regression was used to identify parametric correlations, allowing us to evaluate the pandemic’s impact the patients who underwent conization.

## 3. Results

In this descriptive, retrospective study conducted in the Obstetrics-Gynecology Clinic of the Municipal Emergency Clinical Hospital of Timisoara, 404 patients with indication for conization admitted in the 1 April 2018–31 March 2022 time interval was included. The study was split between a pre-pandemic two-year period and a pandemic two-year period. The pre-pandemic period was considered to be between 1 April 2018 and 31 March 2020, with 194 patients, while the pandemic period included the time interval between 1 April 2020 and 31 March 2022, with 210 patients. For determining the pre-pandemic and pandemic periods, we took into consideration the official date on which the pandemic was declared in our country, 16 March 2020.

In the pre-pandemic period, according to Table 1, the highest proportion was observed in the age group of females of reproductive potential, aged 30–39, in 31.95% (62 patients), generally causing a multi-cause infertility problem such as sexually transmitted disease and pelvic inflammatory disease sequelae. Immediately close to that were patients aged 40–49 years, in a proportion of 27.31% (53 patients) in the perimenopausal period. The next age group with a significant percentage that presented in the clinical situation ranged from 20–29 years, with a percentage of 22.16% (43 patients), the age group of the acquisition of sexually transmitted infections, including HPV. From the age of 50 years, the age from which cancer pathology generally begins, a gradual decrease in the percentages was observed, in the following order: age group 50–59 years, with a percentage of 14.43% (28 patients); age group 60–69 years, with a percentage of 3.60% (7 patients); and age group 70–79 years, with a low frequency of 0.51% (one patient).

In Table 1 in the pandemic period, patient distribution was observed in age groups similar to the pre-pandemic period, namely 30–39 years with a percentage of 34.28% (72 patients), 40–49 years with a percentage of 31.90% (67 patients), 20–29 years with a percentage of 20.47% (43 patients), 50–59 years with a percentage of 9.52% (20 patients), 60–69 years in 3.33% (7 patients), and 70–79 years with the lowest percentage, 0.47% (1 patient).

There was a statistically significant age difference between the two study periods, both by the average age of patients and by age group. The mean difference was 32 years before the pandemic and 35 years during the pandemic (*p*-value > 0.05). The majority of patients presenting for investigations before and during the pandemic were in the 30- to 39-year-old age group (31.95%, respectively; 34.2% *p*-value = 0.003). The biggest patient loss ratio identified by age group was in the 50–59-year group, with 14.53% in the pre-pandemic period and 9.1% in the pandemic period. From Table 2, 50.51% (98 patients) of rural patients were observed out of all patients who presented in the clinic during the pre-pandemic period, compared with 49.48% (96 patients) of urban patients.

Table 2 shows that, compared to the pre-pandemic era, the pandemic period had a larger percentage of patients in urban settings of 60.47% (127 patients). During the pandemic era, patients from rural regions presented in the clinical study at a lower rate of 39.52% (83 patients), as seen in Table 2.

**Figure 1:**
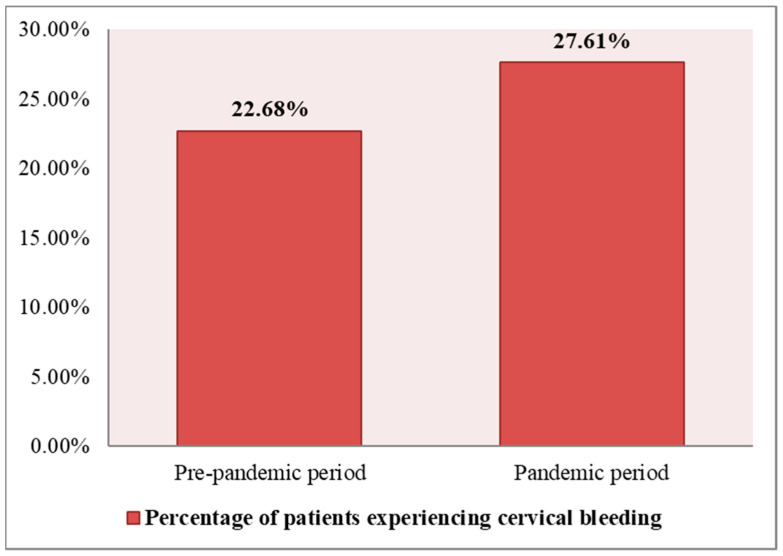
Percent distribution of patients experiencing cervical bleeding.

A higher percentage of patients experiencing cervical bleeding as a clinical manifestation in the pandemic period vs. the pre-pandemic period was observed based on Table 3 and Figure 1. Cervical bleeding was present pre-pandemic in 22.68% of patients (44 out of 194 patients), and during the pandemic, it was present in 27.61% of patients (58 out of 210 patients).

**Figure 2:**
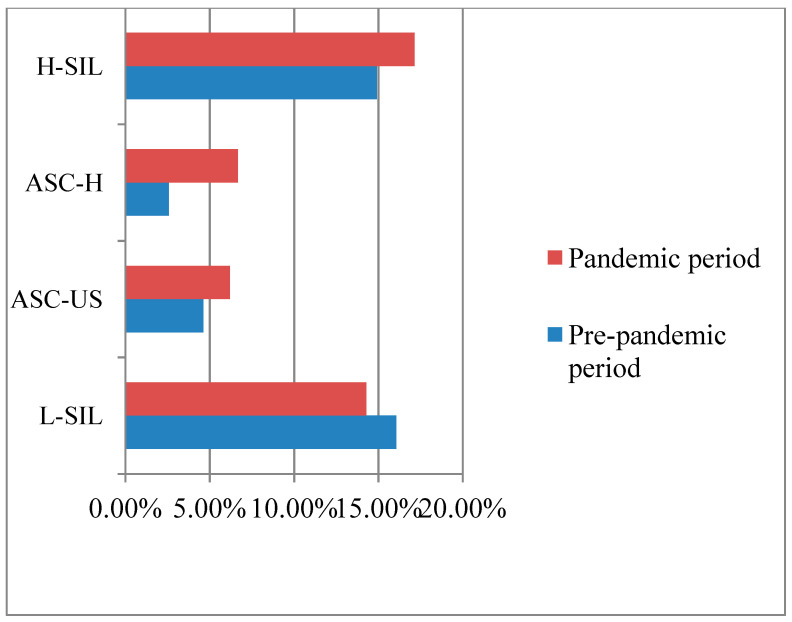
Percentage distribution of PAP test results over the two periods analyzed.

In the pre-pandemic period, Figure 2 shows the high incidence of low-grade intraepithelial lesions (L-SIL), with 16.06% (31 patients) diagnosed with this dysplastic change in the cervical epithelium immediately followed by high-grade intraepithelial lesions (H-SIL) with 14.90% (29 patients). In a lower number, atypical squamous cell pap cytology was found, with more frequent undetermined squamous atypia (ASC-US), which lends itself to differential diagnosis with low-grade intraepithelial lesions (L-SIL), with a percentage of 4.63% (nine results). A percentage of 2.57% (five patients) had squamous types that could not exclude an H-SIL lesion (ASC-H), for which the differential diagnosis with H-SIL was made.

During the pandemic period, a high incidence of H-SIL lesions was seen, according to Figure 2, with 17.14% (36 patients) having this result. The next lesion right after was L-SIL, with 14.28% (30 patients) diagnosed. The percentage of patients with squamous disease was close between the two categories in this class, namely 6.19% (13 patients) had ASC-US lesions and 6.66% (14 patients) had ASC-H results.

**Figure 3:**
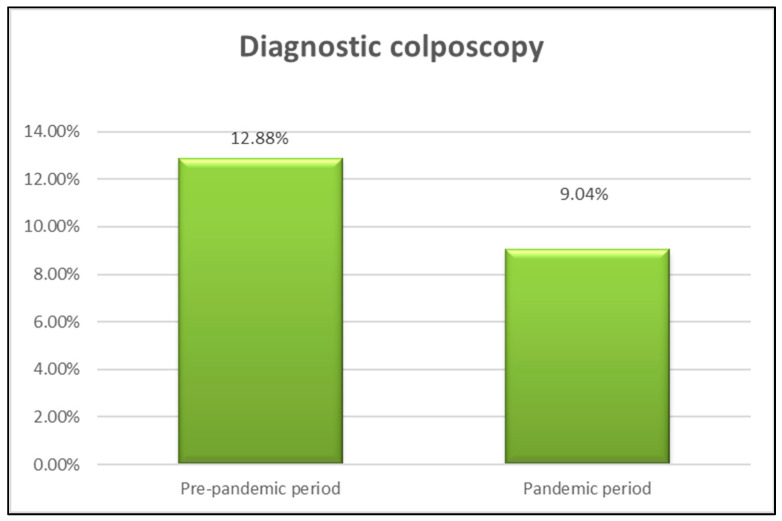
Percent distribution of patients with colposcopy examination performed in the two periods of the study.

Based on the data observed analyzing the numerical and percent distribution of patients undergoing colposcopy examination, a total of 12.88% of patients underwent diagnostic colposcopy in the period prior to the pandemic, namely 25 out of 194 patients, and, in the pandemic period, 9.04% of patients (19 out of 210 patients). Figure 3 shows a slightly increased proportion of patients with colposcopy pre-pandemic (12.88%) versus pandemic (9.04%). Using the colposcopy examination, cervical lesions can be highlighted as aceto-white and iodine-negative, with different characteristics depending on the degree of the lesion.

A dual immunocytochemical signal confirms cell cycle dysregulation caused by changes in the cell genome and signals the potential for progression to a high-grade cervical lesion. According to Table 4, a small fraction of 1.54% of patients (3 out of 194 patients) were tested prior to the pandemic for cellular markers of infection (identification of double cytology p16 and Ki67). In a higher percentage, 4.28% of patients (9 out of 210 patients) were observed to undergo dual staining (double immunocytochemical staining) for the detection of infection cellular markers in the pandemic period.

**Figure 4:**
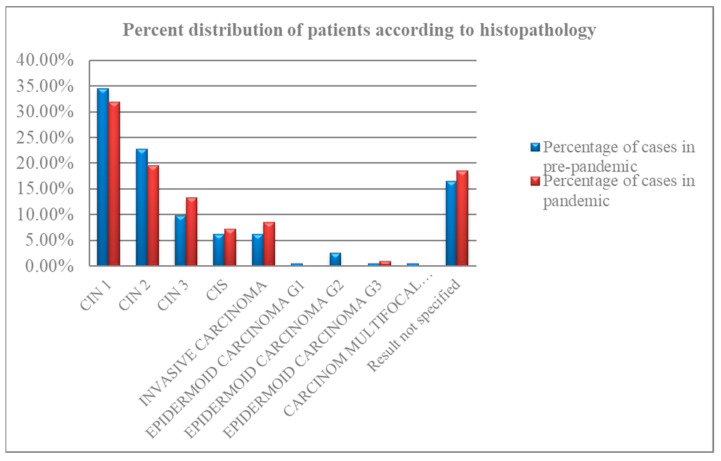
Distribution of patients by histopathology outcome in the pre-pandemic and pandemic periods.

The number of individual tests for cervical cancer was significantly decreased during the pandemic by the percentage of pap smears, HPV tests, and colposcopies, but we noticed that the CIN tests increased. This was due to the attempt to manage, noninvasively and without hospitalization, the lesions of the cervix in the pandemic period, postponing the biopsy or conization of the cervix; this has led to a delay in the diagnosis of certainty. From the data presented in Table 5 and Figure 4 on the numerical and percentage distribution of patients by pre-pandemic histopathology results, the highest incidence was observed for CIN 1 lesion in 34.53%, or 67 cases. The next commonly diagnosed lesion, in 22.68% (44 patients), was the CIN 2 lesion. Moreover, CIN 3 was observed in 9.79%, corresponding to severe dysplasia in 19 patients. In situ carcinoma (CIS) and invasive squamous cell carcinoma were diagnosed in equal numbers, 12 patients, and an equal percentage, 6.18%. In a small percentage of 0.51% (one patient), epidermoid carcinoma G1, epidermoid carcinoma G3, and keratinized multifocal carcinoma were detected separately in the histopathology. G2 epidermal carcinoma was diagnosed in 2.57% (five patients). For 16.49%, representing 32 patients, the outcome of the specified histopathologic examination was not found.

From the data observed in Figure 4 and Table 6 analyzing the numerical and percentage distribution of patients by histopathology results in the pandemic period, the most frequently diagnosed CIN 1 lesion was evident in 31.90% (67 patients). This was followed by CIN 2 with 19.52% (41 patients) and CIN 3 with 13.33% (28 patients). The incidence of in situ carcinoma (CIS) and invasive carcinoma was observed in close proportions: 7.14% (15 patients) and 8.52% (18 patients), respectively. G3 epidermal carcinoma was present in 0.95%, or two patients. A total of 18.57% (39 patients) had unspecified histopathology. Similar concerning findings were observed in the stage of newly diagnosed cancers, with a significant difference in CIN 3, CIS, invasive carcinoma, and G3 cancers of 8% more during the pandemic.

A noteworthy statistically significant finding was identified between low-grade squamous intraepithelial lesions (L-SIL) and higher cervical intraepithelial neoplasia (CIN 3). The analysis revealed a positive correlation of rho = 0.256 (*p* = 0.009) (Table 6). Another significant finding was between cervical bleeding and CIN 3 (rho = 354, *p*-value = 0.004).

## 4. Discussion

The primary impact of the COVID-19 pandemic appears to be a decrease in the number of screening appointments and diagnostic procedures, resulting in the delayed diagnosis and treatment of cancer patients.

Prior studies have reported a significant decrease of 57.21% in the number of cases diagnosed with high-grade dysplasia in the pandemic period, compared to our study, where we observed an 8% increase in the diagnosis of high-grade dysplasia, or CIS, invasive carcinoma in the pandemic period. In a similar way, the number of colposcopies performed was observed in both studies to be decreasing in the pandemic period compared to the pre-pandemic period [26].

Also, a considerable decrease in the number of cervical cancer screening tests and the number of newly diagnosed patients with cervical cancer have been described in previous studies. Cervical cancer investigations were significantly affected by a mean percent change of 49% in reduction of test volume over the two pandemic years (*p*-value < 0.001) [27].

Similar findings were confirmed by a study taking place in Brasilia, Sao Paolo, which indicated that the percent of cases positive for CIN-1 (*p* < 0.0410) and CIN-3 (*p* < 0.0012) increased in 2020 and 2021 as compared to 2019 levels [28].

A significant decrease in the quantity of pap smears, conizations, and mammograms performed since the start of the COVID-19 epidemic was noted in another study carried out by Mateus BO. Duarte. In addition, stage I and II breast cancer adjuvant treatment presented a reduced realization rate, whereas palliative treatment delivered for advanced cervical cancer increased [29].

To prevent the spread of COVID-19 and shift the medical resources to emergency areas [20,30,31], it is necessary to cancel elective surgeries in all hospitals [20,32]. WHO has implemented new protocols to provide safety for patients and doctors, continuing to restrict surgical interventions to emergencies only [20,33,34].

The last data from the nationwide Netherlands Cancer Registry showed that there was a notable decrease in overall cancer diagnoses when compared to the pre-pandemic period year of 2020 [18,35]. Similar data have been reported for Denmark, Germany, Austria, Poland, the United States, and the United Kingdom [18,36,37].

The most concerning potential adverse effect of the disruption of cancer screening programs could delay the diagnosis of tumors, causing a shift to diagnosis at more advanced stages and fewer treatment options, resulting in a worse prognosis [38,39,40,41,42,43] and an increase in future cancer morbidity and mortality [44].

Because of a break in cervical screening service at the beginning of the pandemic, four studies showed that they expected an increased diagnosis rate of advanced and invasive lesions of cervical cancer or deaths in the upcoming years due to the lack of hospitalizations of patients and implicitly by decreasing curative surgical maneuvers [45,46].

The incidence of cervical cancer is expected to increase if compensatory screening programs are not implemented as soon as possible to recover the diagnosis of all cases of cervical dysplasia omitted during the pandemic period by non-testing time. On the assumption that healthcare services will not be able to increase the detection rate for cervical lesions to a level even higher than in previous years, it will become difficult to lower the cervical cancer incidence. Therefore, there will be a delay in the diagnosis of cervical cancer for women to undergo an examination (25–64 years) according to standard international protocols [47,48,49].

A study by Smith et al. [50] suggested the number of diagnoses of cervical cancer would increase by 1.1–3.6% due to the discontinuity of cervical cancer screening in 2020 [44]. In another study, Gupta et al. [51] noted an increase from 2.52% to 3.80% in cervical cancer-related deaths, with treatment delays ranging from 9 weeks to 6 months [45], while Kregting et al. [52] projected a twofold increase in deaths from cervical cancer per 100,000 people in 10 years [46]. Davies et al. [53] estimated that in the next 3 years, there will be a considerable increase in newly diagnosed cases of cervical cancer [47].

The WHO had demonstrated the need to increase spending on prevention and screening tests, particularly in low-financial countries, and this becomes even more urgent during post-pandemic times when additional financial resources are required to cover the missing tests [42,54].

Another solution for effectiveness could be to prioritize those who are most likely to develop cancer, such as women with a personal or family history of breast cancer, ovarian cancer, women with HIV immunodeficiencies, or women who have not been in screening for several years [42,55,56].

The systematic review of Ferrara et al. [57] conducted on seven studies observed reductions in HPV vaccine uptake and coverage during COVID-19 [51]. Reports of cervical cancer screening and cancer diagnostic activities have shown a major impact of the pandemic on access to screening services and diagnostic procedures. All but one of the studies investigating the treatment of cervical cancer reported changes in the number of women with cervical lesions receiving treatments and delays and discontinuations of treatment [57]. Also, in the context of the COVID-19 pandemic, HPV vaccination and routine immunization services have presented significant disruptions, with decreases in immunization coverage [57,58,59].

In our study, the proportion of patients with diagnostic colposcopies was higher in the pre-pandemic period, at 12.88%, but tests for the identification of double cytological markers p16 and Ki67 increased during the pandemic period, reaching 4.28%.

Cervical cancer screening plays an important role worldwide in the early diagnosis and high rates of cervical cancer cure. However, the COVID-19 pandemic required several drastic measures to halt the spread of the new coronavirus, SARS-CoV-2, limiting access to non-invasive and invasive investigations essential for cervical cancer diagnosis.

The COVID-19 pandemic has shown an impact on both pregnant and non-pregnant females of reproductive age. Several studies have reported increased mortality rates and poor maternal and neonatal outcomes among susceptible populations, such as oncological patients, pregnant women, and newborn infants [60,61,62]. In pregnant patients, the most common symptoms at admission to hospital were fever and cough, and pregnant patients were more likely to be admitted to intensive care units compared to the non-pregnant patients. Allotey et al. performed a meta-analysis on 435 studied including a total of 926,232 women and concluded that several risk factors, such as preexisting morbidities (diabetes, hypertension, obesity), increased maternal age, and non-white ethnicity, were associated with severe outcomes [63].

The pandemic context, avoiding hospital contact to minimize exposure to SARS-CoV-2, and the cessation of hospitalizations and non-urgent interventions for COVID, led to delays in screening tests for cervical cancer and biopsies for histopathological certainty. As consequence, there was an increase in the pandemic period compared with the pre-pandemic period of the cervical assessment tests that can be performed in outpatient service without admission, like dual-staging for p16/ki-67. Careful monitoring of the downstream impacts of COVID-19-related service disruptions on cervical pre-cancer and cancer outcomes will be critical for assisting service providers in planning and mitigating negative implications. Based on these findings, we predict similar future outcomes in our area, and future priority measures for catch-up should be developed to balance potential resource constraints with clinical demands.

This study explored the association between cervical cancer and the transmission dynamics of SARS-CoV-2. It reported a notable increase in cervical bleeding as a primary reason for medical consultations during the pandemic period, suggesting potential impacts of the pandemic on the presentation of gynecological symptoms. Additionally, a key contribution of this research was the identification of a higher degree of cervical lesions observed during the same period. This finding may indicate altered healthcare access or changes in patient behavior in response to the pandemic, warranting further investigation into its causes and implications for public health.

Several limitations of the current study include its single-center design and the availability of data from patient records. Data collected from a single site may not be representative of the overall Romanian population, as patient background factors might vary significantly across the nation.

## 5. Conclusions

The conclusions of our study articulate that cervical cancer, a malignancy with significant preventive potential, has been impacted by the COVID-19 pandemic due to disruptions in screening and medical checks, although patient addressability in our study remained largely stable. We observed an increase in consultations for cervical bleeding during the pandemic and a notable decrease in appointments among the 50–59 age bracket and patients from rural areas. However, while previous research has reported a significant decrease in the number of cases diagnosed with high-grade dysplasia in the pandemic period, our results showed an 8% increase in the diagnosis of high-grade dysplasia, or CIS, invasive carcinoma in the pandemic period. Our data, while reflecting regional specificities, underscore the importance of prioritizing vulnerable sub-groups in cervical cancer screening during pandemic situations. To aid public health measures, enhancing infrastructure and resilience is essential, incorporating strategic goals to support sustained cancer services amidst and in the aftermath of global emergencies.

## Figures and Tables

**Table 1:** Patient distribution by age group.

Age (Years)	Pre-Pandemic Period (n = 194)	Pandemic Period (n = 210)	*p*-Value
20–29	43 (22.16%)	43 (20.47%)	0.716
30–39	62 (31.95%)	72 (34.28%)	0.673
40–49	53 (27.31%)	67 (31.90%)	0.328
50–59	28 (14.43%)	20 (9.52%)	0.166
60–69	7 (3.60%)	7 (3.33%)	0.998
70–79	1 (0.51%)	1 (0.47%)	0.998

Data analyzed with Fisher’s exact test.

**Table 2:** Patients’ distribution by place of origin.

Place of Origin	Pre-Pandemic Period (n = 194)	Pandemic Period (n = 210)	*p*-Value
Urban	96 (49.48%)	127 (60.46%)	0.716
Rural	98 (50.51%)	83 (39.52%)	0.673

Data analyzed with Fisher’s exact test.

**Table 3:** Incidence of cervical bleeding.

	Pre-Pandemic Period		Pandemic Period	
	Number of patients	Percent	Number of patients	Percent
Cervical bleeding	44	22.68%	58	27.61%
Total	194	100%	210	100%

**Table 4:** Distribution of patients who were immunohistochemically tested.

	Dual Staining Tests	Total Number of Patients	Percent of Total Patients	*p*-Value
**Pre-pandemic period**	3	194	1.54%	
**Pandemic period**	9	210	4.28%	

**Table 5:** Distribution of patients by histopathology during the pre-pandemic period.

Histopathology Results of Cervical Samples	Cases in the Pre-Pandemic Period (n = 194)	Cases in the Pandemic Period (n = 194)	*p*-Value
CIN 1	67 (34.53%)	67 (31.90%)	0.998
CIN 2	44 (22.68%)	41 (18.52%)	0.806
CIN 3	19 (9.79%)	28 (13.33%)	0.213
CIS	12 (6.18%)	15 (7.14%)	0.691
Invasive carcinoma	12 (6.18%)	18 (8.52%)	0.342
Epidermoid carcinoma G1	1 (0.51%)	0 (0.0%)	0.998
Epidermoid carcinoma G2	5 (2.57%)	0 (0.0%)	0.061
Epidermoid carcinoma G3	1 (0.51%)	2 (0.95%)	0.998
Keratinized multifocal carcinoma	1 (0.51%)	0 (0.0%)	0.998
Result not specified	32 (16.49%)	39 (18.57%)	0.431

Data analyzed with Fisher’s exact test.

**Table 6:** Correlation matrix.

	Age	Place of Origin (Urban/Rural)	Cervical Bleeding	L-SIL	H-SIL	CIN 1	CIN 2	CIN 3
**Age**	1.0, *p* = 0.0							
**Place of Origin**	0.062, *p* = 0.752	1.0, *p* = 0.0						
**Cervical Bleeding**	0.293, *p* = 0.107	−0.032, *p* = 0.940	1.0, *p* = 0.0					
**L-SIL**	−0.214, *p* = 0.071	−0.091, *p* = 0.466	0.224, *p* = 0.537	1.0, *p* = 0.0				
**H-SIL**	−0.311, *p* = 0.54	−0.032, *p* = 0.808	−0.021, *p* = 0.814	−0.125, *p* = 0.793	1.0, *p* = 0.0			
**CIN 1**	−0.218, *p* = 0.69	−0.107, *p* = 0.506	0.066, *p* = 0.594	−0.125, *p* = 0.687	0.625, *p* = 0.689	1.0, *p* = 0.0		
**CIN 2**	0.104, *p* = 0.875	−0.076, *p* = 0.406	−0.092, *p* = 0.447	0.152, *p* = 0.623	−0.106, *p* = 0.359	−0.055, *p* = 0.899	1.0, *p* = 0.0	
**CIN 3**	−0.217, *p* = 0.765	0.091, *p* = 0.532	0.354, *p* = 0.004	0.256, *p* = 0.009	−0.152, *p* = 0.066	−0.107, *p* = 0.737	−0.161, *p* = 0.3614	1.0, *p* = 0.0

## Data Availability

The data presented in this study are available on request from the principal author. Data are contained within the article.
